# Maresin 1 resolves aged-associated macrophage inflammation to improve bone regeneration

**DOI:** 10.1096/fj.202001145R

**Published:** 2020-08-14

**Authors:** Rong Huang, Linda Vi, Xiaohua Zong, Gurpreet S. Baht

**Affiliations:** 1 Duke Molecular Physiology Institute, Duke University, Durham, NC, USA; 2 Department of Orthopaedic Surgery, Duke University, Durham, NC, USA; 3 Division of Physical Medicine and Rehabilitation, University of Toronto, Toronto, Canada; 4 Department of Pathology, Duke University, Durham, NC, USA

**Keywords:** bone, fracture healing, inflammaging, macrophage, osteoblast

## Abstract

Inflammaging is associated with poor tissue regeneration observed in advanced age. Specifically, protracted inflammation after acute injury has been associated with decreased bone fracture healing and increased rates of nonunion in elderly patients. Here, we investigated the efficacy of using Maresin 1 (MaR1), an omega-3 fatty acid-derived pro-resolving agent, to resolve inflammation after tibial fracture injury and subsequently improving aged bone healing. Aged (24-month-old mice) underwent tibial fracture surgery and were either treated with vehicle or MaR1 3 days after injury. Fracture calluses were harvested 7 days, 14 days, 21 days, and 28 days after injury to investigate inflammatory response, cartilage development, bone deposition, and mechanical integrity, respectively. Healing bones from MaR1-treated mice displayed decreased cartilage formation and increased bone deposition which resulted in increased structural stiffness and increased force to fracture in the later stages of repair. In the early stages, MaR1 treatment decreased the number of pro-inflammatory macrophages within the fracture callus and decreased the level of inflammatory biomarkers in circulation. In tissue culture models, MaR1 treatment of bone marrow-derived macrophages from aged mice protected cells form a pro-inflammatory phenotype and induced an anti-inflammatory fate. Furthermore, the secretome of MaR1-treated bone marrow-derived macrophages was identified as osteoinductive, enhancing osteoblast differentiation of bone marrow stromal cells. Our findings here identify resolution of inflammation, and MaR1 itself, to be a point of intervention to improve aged bone healing.

## 1 | INTRODUCTION

“Inflammaging” is a term used to describe the chronic low-grade elevation of inflammatory signals observed in advanced age throughout the circulation and in various organs. Chronic increase in the number of inflammatory cells and the amount of inflammatory cytokines has been linked to dysfunctional cellular differentiation and dysregulated matrix production at the site of tissue injury.^1,2^ This phenomenon has been identified as a potential cause of the dysregulated tissue repair and decreased regeneration capacity after acute injury in advanced age.^3,4^

Bone fracture healing is one regenerative process hindered by inflammaging.^5–7^ Disruption of vessels typically leads to the formation of a hematoma and the recruitment of inflammatory cells shortly after a fracture; subsequently, a cartilaginous soft callus forms, followed by a bony callus and finally remodeling of the bony callus to bone occurs.^8^ While the initial inflammatory phase of bone repair is necessary for recruitment of hematopoietic cells and skeletal progenitor cells, it is prolonged in advanced age and considered to be a component of inflammaging. This causes dysfunctional differentiation of progenitor cells and decreased osteoblast activity, eventually impeding proper bone deposition and repair.^9,10^ Clinically, this translates to an increased need for surgical intervention and revision after fracture in the geriatric population.^5–7^ Our study investigates targeting this protracted inflammation as a way to improve bone regeneration.

In our published work, engrafting young hematopoietic cells into aged mice by parabiosis or bone marrow transplantation improved aged fracture healing; osteogenic differentiation of aged progenitor cells was enhanced by signals secreted young macrophages.^11,12^ The importance of macrophage-osteoblast signaling in bone repair has only recently emerged and has yet to be explored as a therapeutic strategy targeting bone repair.

Resolution of inflammation is a highly complicated process involving numerous secreted molecules. Omega-3 fatty acid-derived specialized pro-resolving mediators, often referred to as “SPMs,” are one set of molecules which help orchestrate resolution of inflammation.^13^ A member of this group is the macrophage mediator in resolving inflammation or Maresin 1 (MaR1; 7R,14S-dihydroxy-4Z,8E,10E,12Z,16Z,19Z-docosahexaenoic acid).^14^ MaR1 is synthesized endogenously by macrophages and acts in an autocrine and paracrine fashion to decrease macrophage-associated inflammation. It has been shown to accelerate tissue regeneration in planaria after head resection and to abate neurogenic inflammation secondary to injury in mammals.^15–21^ A role for MaR1 in bone healing has yet to be investigated. Here, we postulate that administration of MaR1 after fracture will resolve age-associated protracted inflammation and subsequently improve bone fracture healing.

## 2 | MATERIALS AND METHODS

### 2.1 | Mouse models

All protocols were approved by the Duke Institutional and Animal Care and Use Committee. Aged mice were purchased from Jackson Labs (C57BL/6J—stock No. 000664) and aged to 24 months. For all experiments, equal numbers of male and female mice were used.

### 2.2 | Maresin 1

MaR1 (7R,14S-dihydroxy-4Z,8E,10E,12Z,16Z,19Z-docosahexaenoic acid) was purchased from Cayman Chemical. In mouse models, MaR1 was injected intraperitoneal at 5 μg/kg and in culture, MaR1 was used at a final concentration of 3.6 ng/mL. These concentrations have been previously proven to be efficacious in modulating inflammatory response.^22^

### 2.3 | Tibial fracture surgery

Fractures were performed as previously described.^23,24^ Briefly, mice were anesthetized and the surgical area proximal to the knee was shaved and disinfected. Following an incision, a hole was drilled into the tibial plateau and a 0.7 mm stainless steel pin was placed into the medullary cavity and cut flush with the tibial plateau. A tibial fracture was induced mid-shaft using blunt scissors and the incision was closed using wound clips. For analgesia, 0.5 mg/kg buprenorphine-sustained release was administered subcutaneously at the beginning of the procedure. MaR1 treatment was administered either at the time of fracture surgery or 3 days postinjury. Fracture calluses were then harvested either 7-, 14-, 21-, or 28-days post injury.

### 2.4 | Analysis of fracture callus

Fracture calluses were dissected and fixed in 10% Zn-formalin at room temperature for 5 days. μCT analysis was conducted using a Scanco vivaCT 80 (Scanco Medical, Brüttisellen, Switzerland) at a scan resolution of 8 μm. Calluses were scanned 1 mm proximal and 1 mm distal from the fracture site and assessed for total volume (TV) and bone volume (BV) in mm^3^, and ratio of bone volume to total volume (BV/TV). Fixed fracture calluses were decalcified using 12% of EDTA pH 7.4, cleared of EDTA, and embedded into paraffin. Sections were cut at a thickness of 5 μm and stained using Safranin-O/fast green to visualize bone and cartilage. A minimum of five sections were used to conduct computer-assisted histomorphometry analysis and results were presented as an amount relative to the total area of the fracture callus. Tartrate resistant acid phosphatase (TRAP) staining was performed as previously described. TRAP-positive cells were quantified as the percentage of osteoclast surface to bone surface.^23^ iNos and Arg1 immunohistochemistry was performed using respective antibody (AbCam, San Francisco, USA) as per the manufacturer’s instructions. Briefly, histological sections were prepared for immunohistochemistry by washing sections with 0.1% Triton (X-100) for 3 minutes and then, washing three times for 3 minutes in PBS. Sections were blocked in 1% of BSA and all solutions here after contained 0.1% of BSA. Sections were stained with antibody against iNos (1:50 dilution in PBS) or Arg (1:500 dilution in PBS) for 60 minutes at 37°C. Sections were then washed in PBS buffer containing 0.1% Triton (X-100) for 3 minutes and then, three times for 3 minutes in PBS. Sections where incubated with secondary antibody for 20 minutes, washed, and DAB solution was added for 30 seconds at room temperature. Mayer’s hematoxylin was used for counter stain and slides were mounted prior to imaging. ImageJ software was used to quantify stain from imaged sections.

### 2.5 | Mechanical testing

The healing tibiae of mice were harvested 28 days postfracture surgery, wrapped in PBS-soaked gauze and stored at −80°C. In preparation for mechanical testing, tibiae were brought to room temperature by being placed into fresh, room temperature PBS for 2 hours. Samples were tested using four-point bending in the medial-lateral direction with the medial side in tension using an ElectroForce 3220 Series III instrument (TA Instruments, New Castle, USA). Cylindrical rollers 2 mm in diameter were used to apply the four points of loading. The midpoints of the top two rollers were positioned 4 mm apart while the midpoints of the bottom two rollers were positioned 10 mm apart. The top fixture was able to tilt horizontally to allow for simultaneous contact between all four rollers and the sample. To position each tibia within the four-point bending fixture, the most proximal location of the tibia-fibula junction was aligned with the outer edge of one of the bottom loading rollers, ensuring the fracture callus was centered among all rollers. Bending failure tests were performed in displacement control at a rate of 0.025 mm/second. Testing was terminated by failure, as determined by a 95% drop in load.

Load, displacement, and time were recorded at a sampling frequency of 10 Hz. Structural stiffness (N/mm) was calculated as the slope of the load versus displacement data between 30% and 70% of the load at failure, to exclude nonlinear toe-regions in the force-displacement curves. Force to fracture was identified as the maximal load that occurred prior to failure.

### 2.6 | Flow cytometry

Fracture calluses were dissected under a dissection microscope and washed with PBS. Tissue was then cut into approximately 1 mm^2^ × 1 mm^2^ pieces and digested with Collagenase type I (0.2 mg/mL) at 37°C for 2 hours under light agitation. At the end of digestion, cell debris and clumps were removed by passing through a 70 μm cell strainer. Cells were pelleted under centrifugation to remove excess solution and Red Blood Cell Lysis Buffer (Thermo Fisher, Waltham, USA) was added at RT for 5 minutes. Cells were centrifuged and liquid was removed. Cells were then mixed with Flow Cytometry Staining Buffer (Thermo Fisher, Waltham, USA). Primary antibodies were added (CD11b, Ly6c, Ly6G, 1:500) and incubated at 4°C for 30 minutes. Cells were then washed twice with PBS and assessed using an Attune Nxt Flow Cytometer (Thermo Fisher, Waltham, USA). Neutrophil contamination was removed from studied cell populations by only investigating Ly6G negative cells. Subsequently, pro-inflammatory macrophages were then identified as CD11b positive, Ly6c high cells (CD11b+,Ly6C^high^) while anti-inflammatory macrophages were identified as CD11b positive, Ly6c low cells (CD11b+,Ly6C^low^).

### 2.7 | Immunoassays

Inflammatory biomarkers and angiogenic biomarkers were quantified by ELISA. Custom multiplex assays were performed to measure mouse pro-inflammatory markers (IL-1β, IL-6, KC, IL-10, TNFa) and cytokines (MCP-1, MIP1α) (MesoScale Discovery, Gaithersburg, USA) in serum samples (diluted two-fold and four-fold, respectively). Mean reported intra- and inter-assay coefficients of variation for these assays are all <10% and <12%, respectively. Angiogenic markers (VCAM, VEGF) (R&D Systems Inc, Minneapolis, USA) were measured in serum samples and fracture callus tissue lysates (diluted 50-fold and 20-fold for measurement of VCAM, respectively and diluted fivefold for measurement of VEGF). Mean reported intra- and inter-assay coefficients of variation for these assays are all <10% and <12%, respectively.

### 2.8 | Bone marrow-derived macrophage culture

Unfractured mice were euthanized at 24 months of age and the femurs and tibiae were dissected and cleaned of soft tissue. Bone marrow was flushed from the long bones and cell clumps were dissociated using passage though 18G needle. Cells were then plated at a density of 10 × 10^5^/cm^2^ in macrophage media (DMEM, 10% FBS, 100 U/ml Penn/Strep, and 20 ng/mL mCSF). Cells were grown to confluence and then passaged and allowed to adhere overnight. Cultures were then treated with 10 ng/mL of LPS or 40 ng/mL of IL-4 in macrophage media, in the presence or absence of 10 nM MaR1, for 24 hours. At the end of this period, RNA was isolated from lysates and assessed by RT-PCR.

### 2.9 | Conditioned media model

Unfractured mice were euthanized at 24 months of age and the femurs and tibiae were dissected and cleaned of soft tissue. Bone marrow was flushed from the long bones and cell clumps were dissociated using passage though 18G needle. Cells were then plated at a density of 10 × 10^5^/cm^2^ in macrophage media (DMEM, 10% FBS, 100 U/mL Penn/Strep, and 20 ng/mL mCSF). Cells were grown to confluence, and then, passaged and allowed to adhere overnight. Cultures were then treated with vehicle or 10 nM MaR1 in macrophage media. After two days of treatment, cells were washed with PBS and plating media (AMEM, 10% FBS, 100 U/mL Penn/Strep) was conditioned on macrophages for two days and stored at 4°C until use.

### 2.10 | Bone marrow stromal cell culture

Unfractured mice were euthanized at 24 months of age and the femurs and tibiae were dissected and cleaned of soft tissue. Bone marrow was flushed from the long bones and cell clumps were dissociated using passage though 18G needle. Cells were then plated at a density of 500 × 10^3^/cm^2^ in plating medium (AMEM, 10% FBS, 100 U/mL Penn/Strep) for 7 days. Cultures were then passaged and cells were differentiated to osteoblasts in osteogenic medium (AMEM, 10% FBS, 100 U/ml Penn/Strep, 30 μM ascorbic acid, 10–8 M dexamethasone, 8 mM sodium phosphate). After 15 days in differentiation media, wells were washed with PBS, fixed using 10% formalin, and stained for alkaline phosphatase using FastRed (Sigma Inc, St. Louis, USA) or for mineral using 2.5% silver nitrate solution (Von Kossa) on a light box. Replicate wells were washed with PBS and RNA was extracted using TRIzole Reagent (Invitrogen Inc, Waltham, USA) as per manufacture’s protocol.

### 2.11 | Reverse transcription polymerase chain reaction (RT-PCR)

After RNA extraction, purity and quantity of RNA were determined using spectrometric methods. cDNA template was generated using random hexamers. RT-PCR was performed using PowerUp SYBr Green Master Mix (Applied Biosystem, Foster City, CA, USA) and primers were purchased from Applied Biosystems (Foster City, CA, USA). *Alkaline phosphatase (Alp)* (5′→3′) Forward, GACAGGACACACACACACA; Reverse, AAACAGGAGAGCCACTTCA; *Bone sialoprotein (BSP)* (5′→3′) Forward, ACAATCCGTGCCACTCACT; Reverse, TTTCATCGAGAAAGCACAGG; *type I collagen (Col1)* (5′→3′) Forward, CACCCCAATCTGGTTCCCTC; Reverse, CATAAGCCAAGTGGGCAGGA; *Interleukin-1 (IL-1)* (5′→3′) Forward, TGGAGAGTGTGGATCCCAAG; Reverse, GGTGCTGATGTACCAGTTGG; *Nitric oxide synthase (iNOS)* (5′→3′) Forward, CGAAACGCTTCACTTCCAA; Reverse, TGAGCCTATATTGCTGTGGCT; *Interleukin-6 (IL-6)* (5′→3′) Forward, CTGCAAGAGACTTCCATCCAG; Reverse, AGTGGTATAGACAGGTCTGTTGG; *Tumor necrosis factor alpha (TNFa)* (5′→3′) Forward, CTGAACTTCGGGGTGATCGG; Reverse, GGCTTGTCACTCGAATTTTGAGA; *Arginase 1 (Arg1)* (5′→3′) Forward, CAGAAGAATGGAAGAGTCAG; Reverse, CAGATATGCAGGGAGTCACC; *Transforming growth factor beta 1 (TGFb)* (5′→3′) Forward, TGACGTCACTGGAGTTGTACGG; Reverse, GGTTCATGTCATGGATGGTGC. Transcript levels in samples were investigated using a ViiA 7 Real-Time PCR System and compared to the transcript of ribosomal protein 18S as a housekeeping control. A minimum of five replicates of all samples were analyzed.

### 2.12 | Statistical analysis

All statistical analysis was performed using GraphPad PRISM 5 (version 5.01). Data are expressed as mean ± standard deviation. Groups were compared using independent t-tests. Statistical significance was assigned to P values less than 0.05 unless otherwise identified.

## 3 | RESULTS

### 3.1 | MaR1 treatment after injury improves aged fracture healing

**FIGURE 1:**
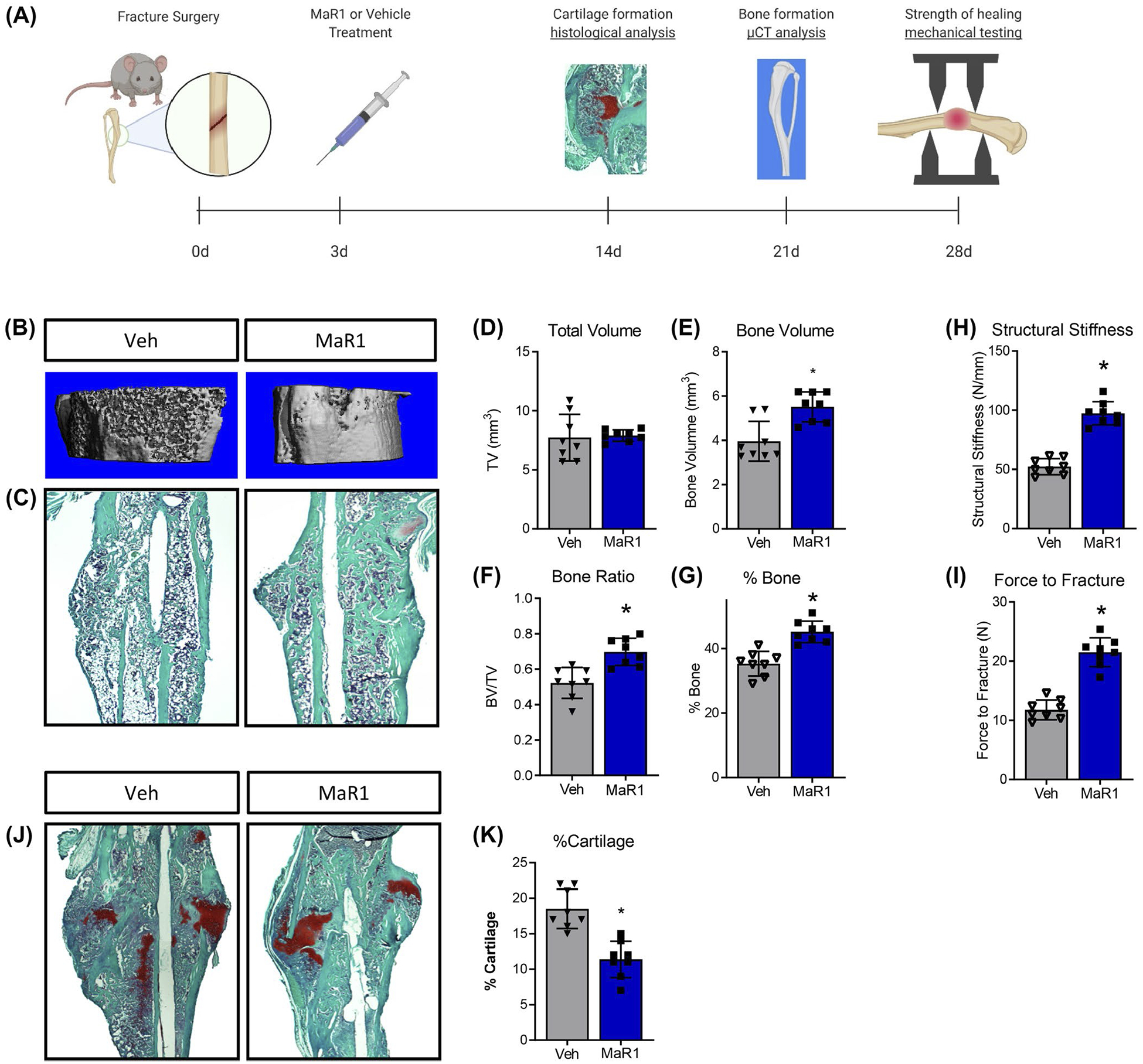
MaR1 treatment of aged mice improves aged fracture healing. A, To test whether MaR1 alters fracture healing, 24-month mice underwent tibial fracture surgery and were treated with either MaR1 or vehicle (veh) 3 days after injury. Bone deposition metrics were assessed 21 day after injury using (B) μCT and (C) subsequently paraffin embedded sections were stained with Safranin-O/fast green (2.5X displayed). μCT was used to determine (D) total volume (TV), (E) bone volume (BV), and (F) bone ratio (BV/TV). G, Histomorphometry of stained sections was used to identify the amount of bone tissue deposited within the fracture calluses. Physical integrity of healed calluses was assessed 28 days post fracture using mechanical testing to determine (H) structural stiffness and (I) force to fracture. J, Cartilage deposition was assessed in 14-day fracture calluses by staining paraffin-embedded sections with Safranin-O/fast green and subsequently using histomorphometry to identify (K) the amount of cartilage deposited within the fracture calluses (2.5X displayed). For all groups, n = 8. Data are expressed as mean ± standard deviation. *P < .05

To first determine the impact of MaR1 treatment on aged bone fracture healing, equal numbers of 24-month-old male and female mice underwent tibial fracture surgery and were treated with either vehicle (Veh) or MaR1 3 days injury. Bone healing was assessed by measuring cartilage deposition, bone deposition, and mechanical strength of the healing tissue at various time points (Figure 1A).

Healing tibiae were harvested at 21 days post-fracture; bone deposition was first assessed by μCT (Figure 1B) then decalcified, paraffin-embedded tissue sections were stained with Safranin-O/fast green to further define tissue deposition within the fracture callus (Figure 1C). Although μCT analysis demonstrated no change in the total volume (TV) of the fracture callus (Veh, 7.7 mm^3^ ± 1.93; MaR1, 7.9 mm^3^ ± 0.94) (Figure 1D), MaR1 treatment increased the bone volume (BV) within the fracture callus (Veh, 4.0 mm^3^ ± 1.84; MaR1, 5.5 mm^3^ ± 1.36) (Figure 1E) and the relative amount of bone within the fracture callus (BV/TV - Veh, 0.50 ± 0.18; MaR1, 0.70 ± 0.15) (Figure 1F). Bone deposition was measured using histomorphometry by identifying light green (fast green positive) staining of collagen matrix as bone. Bone content was higher in MaR1-treated samples, confirming our uCT findings (Veh, 35.25% ± 5.23; MaR1, 45.13% ± 4.18) (Figure 1G).

The structural integrity of healing tibiae was assessed using mechanical testing/four-point bending 28 days after injury. MaR1 treatment increased the structural stiffness (Veh, 52.3 N/mm ± 13.6; MaR1, 97.4 N/mm ± 19.6; 42.8 N/mm) (Figure 1H) and the force to fracture (Veh, 11.8 N ± 3.4; MaR1, 21.5 N ± 4.9) (Figure 1I) of the healed tissue. These findings indicate that MaR1 treatment improved the quality of repaired bone in aged mice.

Cartilage deposition was investigated by paraffin-embedded histological staining (Safranin-O/fast green) of 14-day fracture calluses (Figure 1J). Cartilage deposition (stained red, depicting proteoglycans) was 40% lower in calluses from mice treated with MaR1 than in calluses from vehicle-treated mice (Figure 1K). This time point was also used to investigate osteoclast activity using tartrate-resistant acid phosphatase (TRAP) staining (arrows); no difference was observed (Figure S1). Furthermore, angiogenesis was investigated 7 days after injury by assaying for angiogenic factors, vascular endothelial growth factor (VEGF) and vascular cell adhesion molecule (VCAM); no difference was observed (Figure S2).

Collectively these data indicate that MaR1 treatment after bone injury in aged mice leads to increased bone deposition, increased strength of healed tissue, and overall improved bone healing.

### 3.2 | MaR1 treatment of aged mice alters macrophage phenotype locally and cytokine profile systemically

**FIGURE 2:**
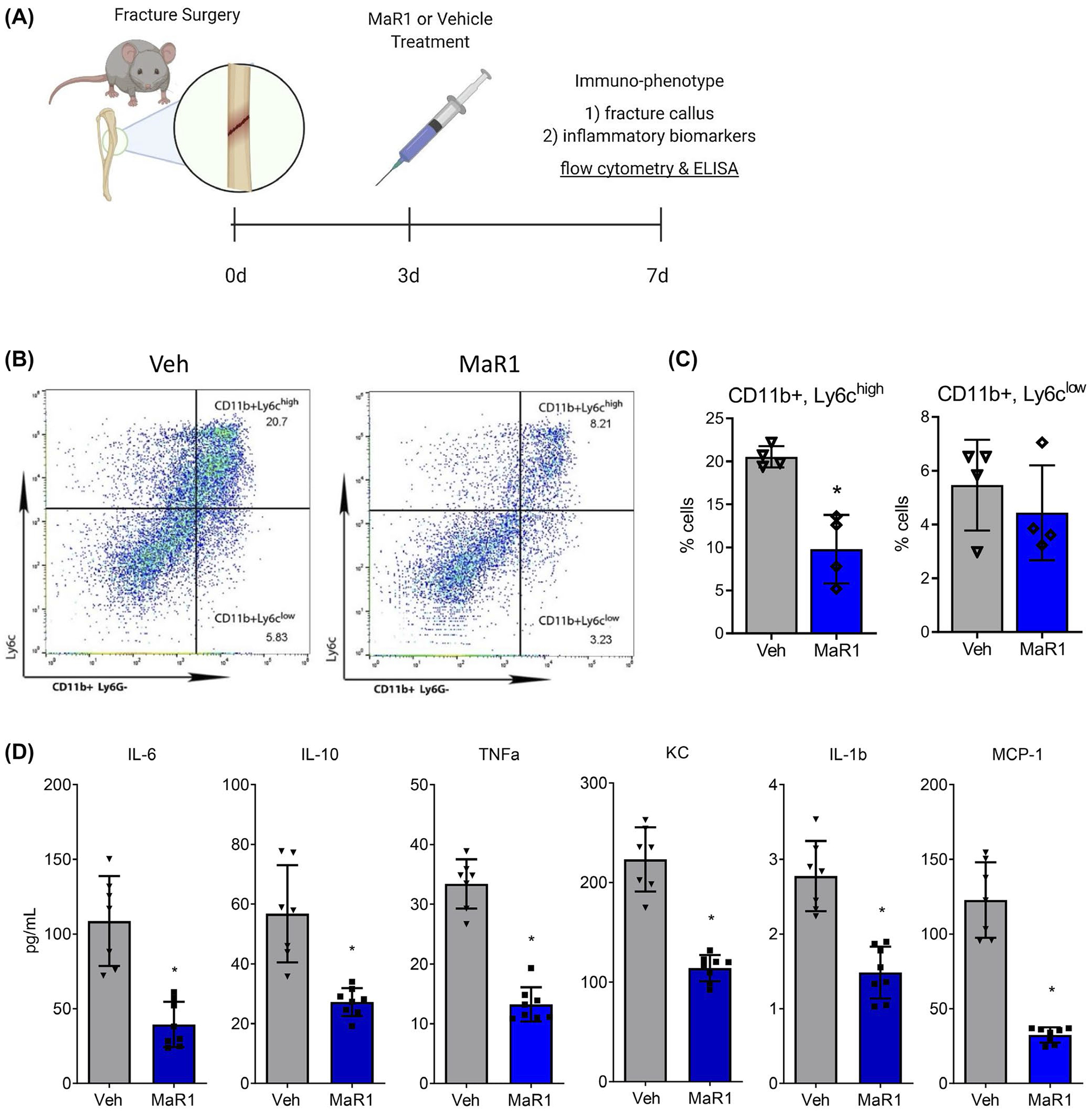
MaR1 treatment decreases the inflammatory profile within the fracture callus and within circulation. A, 24-month old mice underwent tibial fracture surgery and were treated with either MaR1 or vehicle (veh) 3 days after injury. Fracture calluses were isolated 7 days after injury and assessed (B) using flow cytometry to identify (C) the percentage of pro-inflammatory macrophages (CD11b, Ly6G-,Ly6c^high^) and anti-inflammatory macrophages (CD11b, Ly6G-, Ly6c^low^) relative to total cell number with the fracture callus. D, Circulating inflammatory biomarkers in serum were analyzed using ELISA. For flow cytometry, n = 4 per group. For inflammatory biomarkers, n = 8 per group. Data are expressed as mean ± standard deviation. *P < .05

Recent work indicates MaR1 treatment of macrophages in vitro induces production of anti-inflammatory transcripts and proteins, altering the macrophage phenotype to an anti-inflammatory profile.^22^ Here we tested the ability of MaR1 treatment to alter the macrophage phenotype in vivo, during bone fracture healing (Figure 2A). Again, 24-month-old mice underwent tibial fracture surgery and were treated with either vehicle or MaR1 3 days after injury. 7-day fracture calluses were collected and dissected to dissociate cells. Flow cytometry was used to assess macrophage populations within the fracture callus: CD11b was used to identify the leukocyte population; Ly6G was used to remove the neutrophil population; and Ly6c was used to define pro- and anti-inflammatory populations. MaR1 treatment decreased the percentage of CD11b^+^;Ly6G^−^;Ly6c^high^ pro-inflammatory macrophages (Veh, 20.5% ± 1.7; MaR1, 9.8% ± 4.3) while the percentage of CD11b^+^;Ly6G^−^;Ly6c^low^ anti-inflammatory macrophages (Veh, 5.4% ± 1.8; MaR1, 4.4% ± 1.9) was unchanged by treatment (Figure 2B,C). These trends were also observed in immunohistochemically stained sections of 7-day fracture calluses: MaR1 treatment caused a decrease in pro-inflammatory macrophages (stained using iNos) but no change in anti-inflammatory macrophages (stained using Arg1) (Figure S3). Collectively, these findings illustrate a MaR1-induced local immunomodulation within the fracture callus.

Since MaR1 treatment was performed systemically, we used a multiplex ELISA to assess levels of circulating cytokines within the serum of mice, 7 days after fracture injury. MaR1 decreased serum levels of inflammatory cytokines IL-6 (Veh, 108.6 pg/mL ± 29.8; MaR1, 39.4 pg/mL ± 15.8), IL-10 (Veh, 56.8 pg/mL ± 16.3; MaR1, 27.2 pg/mL ± 4.8), TNFa (Veh, 33.4 pg/mL ± 4.1; MaR1, 13.2 pg/mL ± 3.0), KC (Veh, 223.3 pg/mL ± 32.9; MaR1, 114.0 pg/mL ± 13.9), IL-1b (Veh, 2.77 pg/mL ± 0.47; MaR1, 1.48 pg/mL ± 0.35), and MCP-1 (Veh, 122/8 pg/mL ± 24.8; MaR1, 32.4 pg/mL ± 4.7) as determined by ELISA (Figure 2D).

Collectively, these findings show that MaR1 treatment after fracture injury in aged mice causes a decrease in the inflammatory profile both at the fracture callus and systemically, confirming MaR1’s ability to help resolve inflammation after bone injury in aged mice.

### 3.3 | MaR1 treatment at the time of injury is ineffective in modifying inflammation response or bone healing

Interestingly, aged mice treated with MaR1 at the time of fracture did not display this immunomodulation phenotype: there was no change in the number of pro-inflammatory macrophages nor in the number of anti-inflammatory macrophages within the fracture callus; there was no significant change in the levels of inflammatory biomarkers (Figure S4). Furthermore, μCT analysis shows that MaR1 treatment at the time of fracture did not alter any bone healing metrics: cartilage and bone quotient, callus volume, bone volume, structural stiffness, and force to fracture were all similar to vehicle control (Figure S5).

### 3.4 | MaR1 alters macrophage phenotype

**FIGURE 3:**
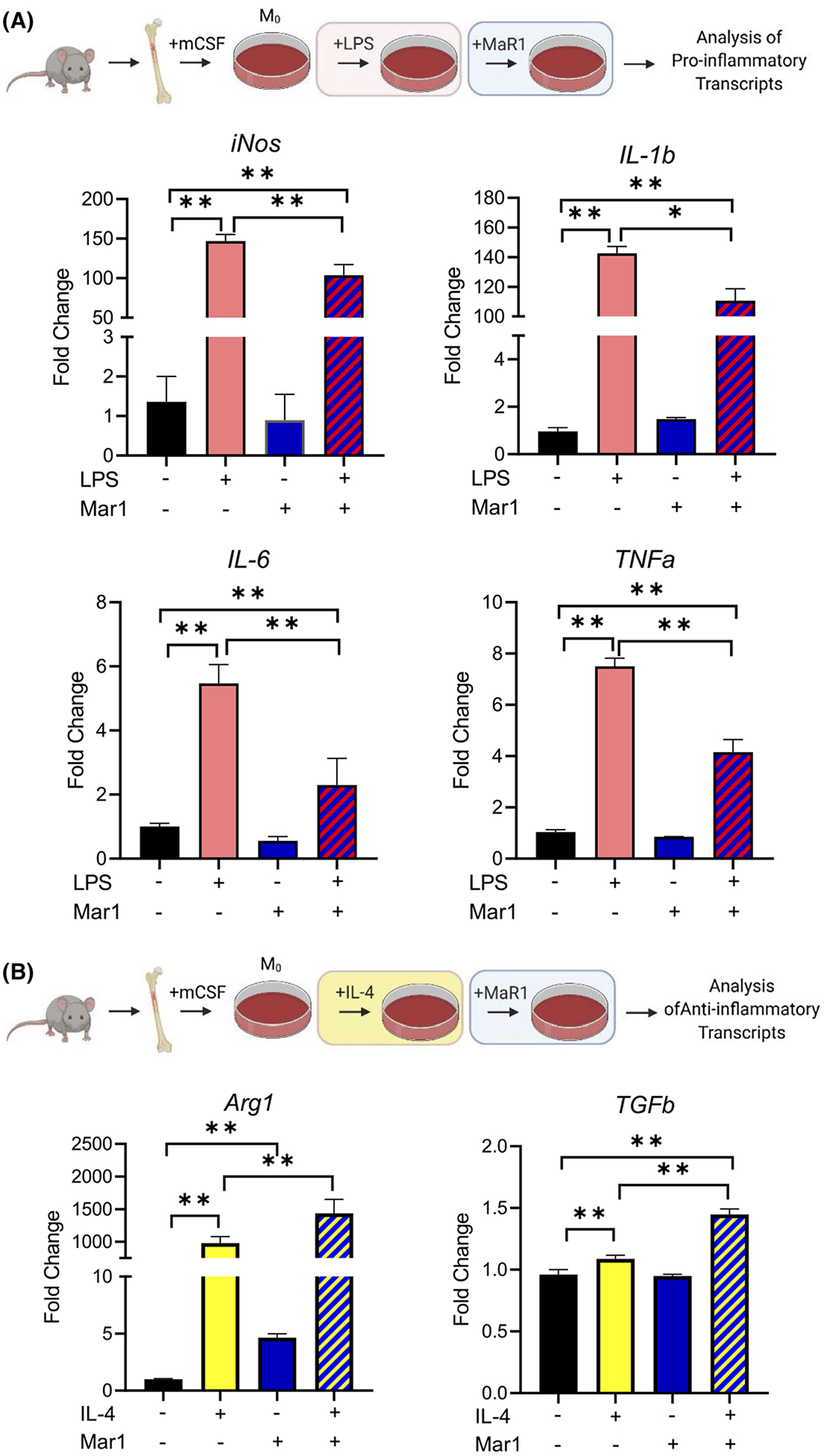
MaR1 induces an anti-inflammatory phenotype in aged bone marrow-derived macrophages. Bone marrow was flushed from 24-month old mice and differentiated into M0 macrophages using mCSF. Cultures were treated with either LPS (10 ng/mL) or IL-4 (40 ng/mL) in the presence of vehicle or MaR1. After 12 hours of treatment, levels of (A) inflammatory transcripts and (B) levels of anti-inflammatory transcripts were assessed using RT-PCR. For all groups, n = 4. Data are expressed as mean ± standard deviation. **P < .05

We have shown that the improved aged bone fracture healing from MaR1 treatment is associated with a change in macrophage phenotype from a pro-inflammatory profile to an anti-inflammatory one. To further characterize this, we turned to tissue culture models. Macrophages derived from the bone marrow of 24-month-old mice were either treated with the pro-inflammatory cytokine LPS (10 μg/mL) or the anti-inflammatory cytokine IL-4 (40 ng/mL) in the absence or presence of MaR1. After treatment, we assessed these cultures for transcript levels of classic markers for pro- and anti-inflammatory macrophages. Predictably, LPS treatment increased expression of the inflammatory transcripts *iNos*, *IL-1b*, *IL-6*, and *TNFa*. MaR1 treatment alone had no effect on these transcripts; however, MaR1 was protective against LPS treatment and decreased LPS-induced transcript production of these inflammatory cytokines (Figure 3A). IL-4 treatment induced transcript expression of the anti-inflammatory macrophage markers *Arg1* and *TGFb*. Interestingly, MaR1 treatment alone recapitulated this trend and had an additive effect when IL-4 and MaR1 were used together (Figure 3B). Collectively, these data indicate that MaR1 has the capacity to act as a macrophage-polarizing agent, altering macrophage cytokine production and shifting macrophage fate toward an anti-inflammatory phenotype.

### 3.5 | MaR1 treatment of aged macrophages induces an osteoinductive secretome

**FIGURE 4:**
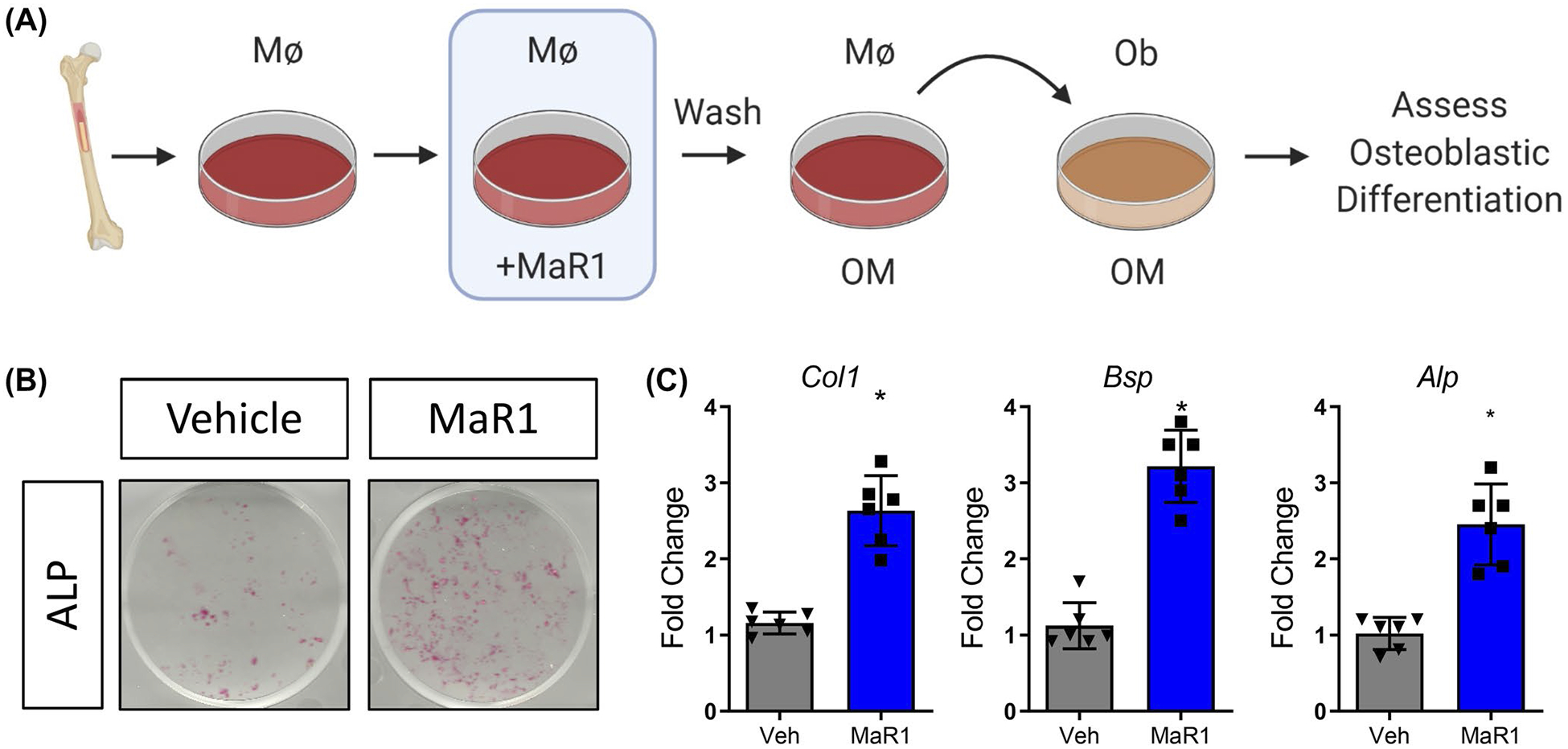
MaR1 induces an osteoinductive secretome in macrophages from aged mice. A, Bone marrow-derived macrophages from aged mice were treated with vehicle or MaR1 and then used to condition osteogenic media. Osteogenic media was subsequently used to differentiate BMSCs from 24-month old mice. After 14 days of differentiation, wells were (B) washed, fixed, and stained for alkaline phosphatase (ALP) or (C) assessed for osteogenic transcripts using RT-PCR. n = 6 per group. Data are expressed as mean ± standard deviation. *P < .05

We demonstrated that MaR1 treatment alters macrophage phenotype to an anti-inflammatory profile resulting in improved aged bone healing. In our previous work, we have shown that macrophage-osteoblast crosstalk is a key component of bone regeneration with the macrophage secretome regulating osteoblast differentiation and activity.^12^ Thus, we designed a conditioned media model to examine the impact of MaR1 treatment of macrophages on the differentiation potential of osteoblasts. First, bone marrow-derived macrophages from aged mice were treated with vehicle or MaR1 and then used to condition osteogenic media. Subsequently, these two preparations of osteogenic media were used to differentiate bone marrow stromal cells (BMSCs) from aged mice to osteoblasts (Figure 4A). Osteoblast differentiation was assessed by alkaline phosphatase (ALP) stain and by measuring osteogenic transcript levels. ALP staining was increased in osteoblast cultures fed by conditioned media from MaR1-treated macrophages (Figure 4B). Likewise, *Col1* transcript levels were 2.4 times higher, *Bsp* transcript levels were 2.9 times higher, and *Alp* transcript levels were 2.4 times higher in osteoblast cultures fed by MaR1-treated macrophage conditioned media (Figure 4C). Importantly, MaR1 had no effect on osteoblast differentiation when directly used within osteogenic media (Figure S6).

**FIGURE 5:**
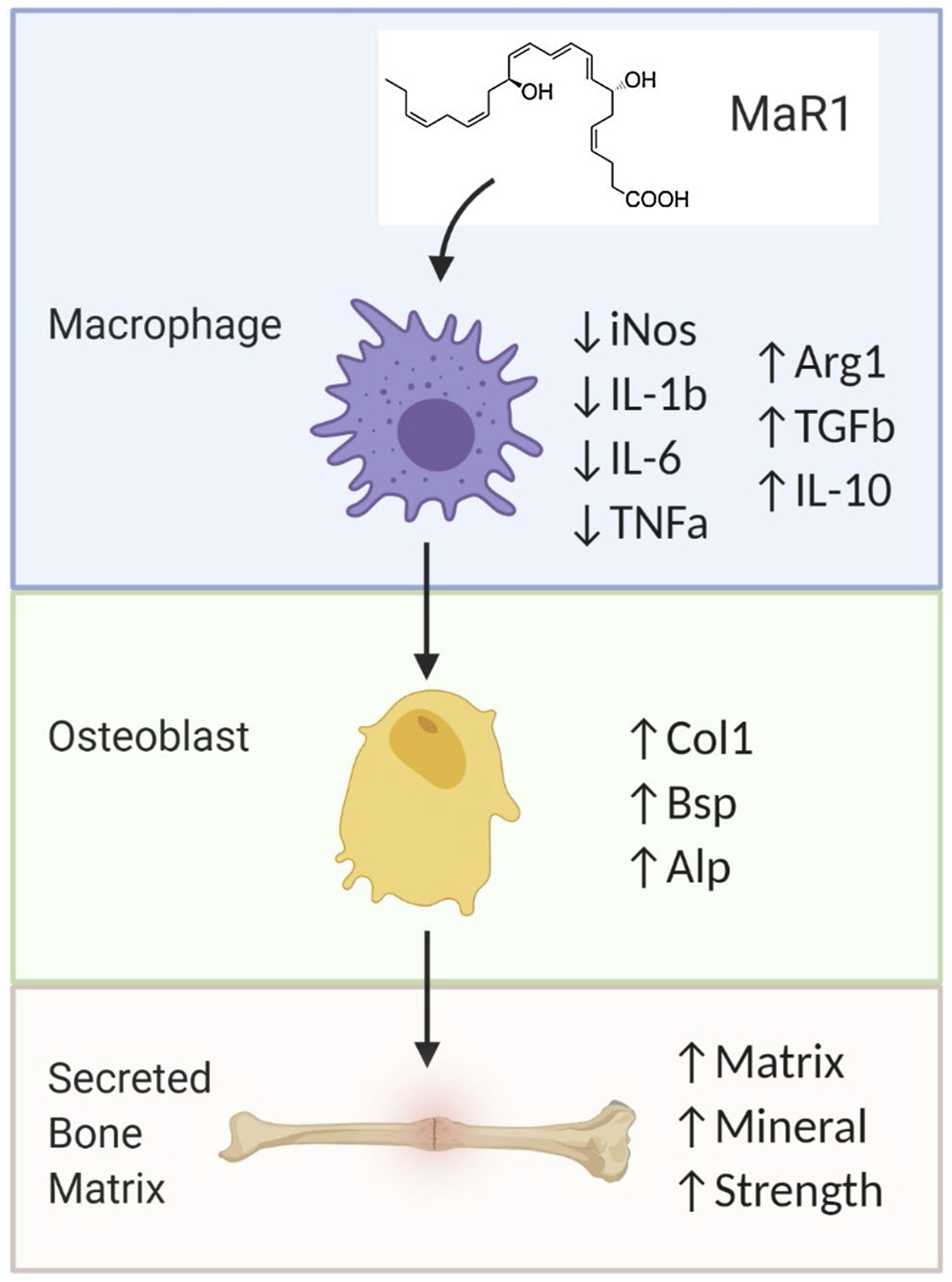
Schematic diagram depicting MaR1-induced improvement of aged bone healing. In aged mice, MaR1 treatment after fracture injury induces an anti-inflammatory phenotype in macrophages at the site of fracture. These macrophages in turn secrete a strengthened reparative (osteoinductive) signal which increases osteoblastic differentiation and activity and subsequently increases bone tissue deposition and improves aged bone regeneration

Collectively, these data point toward a mechanism where aged macrophages treated by MaR1 produce an osteogenic niche that increases the production of bone matrix and subsequently improves bone fracture healing (Figure 5).

## 4 | DISCUSSION

Inflammation during bone repair is necessary for the recruitment of hematopoietic and mesenchymal progenitor cells to the site of injury and normally occurs in a well-regulated and well-timed manner. In advanced age, this inflammatory phase is protracted impeding proper osteoblast differentiation and leading to diminished fracture healing which manifests as delayed bone union, increased rates of re-fracture, failure of orthopedic implants, and nonunion of acute fractures.^1,5–7,10^ Here, we present a potentially therapeutic intervention which involves a reversal of inflammaging shortly after injury by actively shifting pro-inflammatory macrophage signaling to anti-inflammatory macrophage using a small molecule naturally occurring in macrophages.

MaR1 was administered 3 days after fracture surgery and improved bone healing in aged mice, as assessed by bone content and mechanical strength of healed tissue. MaR1 treatment decreased circulating levels of inflammatory biomarkers and decreased the number of pro-inflammatory macrophages within the fracture callus of aged mice. This immunomodulation from a pro-inflammatory to an anti-inflammatory macrophage phenotype is similar to the neural inflammatory model, in which MaR1 treatment decreased microglial activation after fracture surgery,^16,22,25^ though this is the first time it is described in bone. Conventionally, dysregulated biology of anti-inflammatory (alternatively activated/“M2”) macrophages was thought to be the culprit in inflammaging: anti-inflammatory macrophage levels have been shown to positively correlate with angiogenesis and progression of wound healing.^26–28^ Recently however, Clark et al demonstrated that inefficient healing may not be caused by a lack of anti-inflammatory macrophages but rather by a surplus of pro-inflammatory macrophages (“M1”/classically-activated) macrophages.^29^ While more work is required to fully understand age-dependent differences in macrophage response and beyond in immune cell function during regeneration, our work further confirms the relevance of the pro-inflammatory macrophage population when targeting inflammaging to improve tissue repair in advanced age.

Importantly, MaR1 treatment at the time of injury did not alter any metrics of inflammation nor of fracture repair outcome. This was not surprising as MaR1 has been shown to have mild anti-inflammatory ability which likely cannot overcome the strong inflammatory signals released endogenously at the onset of fracture injury. This finding reinforces the importance in the regulation of the inflammatory phase during this initial stage of bone fracture healing. Three days after acute fracture injury is a time point at which inflammation should be dissipating; MaR1 treatment at this stage likely helps to accelerate the endogenous resolution response that is dysregulated with age.^30,31^

In our in vitro model, the secretome of aged macrophages was more osteoinductive when cultures were first treated with MaR1, leading to enhanced differentiation of aged BMSCs to osteoblasts. This echoes our previous findings in which osteogenic media conditioned by bone marrow-derived macrophages from young mice but not aged mice significantly increased the osteogenic potential of aged BMSCs.^12^ Furthermore, we and others have demonstrated that macrophages are required for normal bone healing: when macrophage recruitment was prevented or macrophages were ablated from mice, fracture healing was delayed and nonunion rates were increased.^32–35^ Collectively, this work highlights the importance of understanding the communication between macrophages and osteoblasts during repair.

Formation of the cartilaginous callus is an important stage in bone fracture healing. Interestingly, fracture calluses from vehicle-treated mice contained greater cartilaginous callus ruminants at later time points than did calluses from MaR1-treated mice. While this would seem to indicate increased activation of osteoclasts from MaR1 treatment, TRAP staining within the calluses of both treated and untreated mice was similar. It is important to note that an age-dependent increase in cartilaginous callus has also been seen by others.^29^ This observed increase may be rooted in a failure in trans-differentiation of chondrocytes to osteoblasts,^36–38^ possibly indicating yet to be elucidated implications of communication between immune cells and mesenchymal cells.^39–42^

Age-associated deficits in bone regeneration highlight the need for therapies to enhance repair,^43^ especially considering the numerous shortcomings of current treatment strategies: complications such as improper graft or fusion, heterotopic bone formation, and urologic problems; inconsistent treatment outcomes; invasive surgical administration; and these interventions need to be carried out at the onset of injury.^44–46^ Our work is the first to demonstrate MaR1’s ability to enhance aged bone regeneration. MaR1 treatment was able to combat protracted inflammation after bone injury leading to healing in aged mouse models. MaR1 has been reported to have no known side effects and can be administered systemically after injury, presenting it as a novel therapeutic agent to be used in improving aged bone regeneration.

## Supplementary Material

Supplementary Figures

## Abbreviations:

ALP: alkaline phosphatase
BV: bone volume
BV/TV: bone volume/total volume
IP: intraperitoneal
LPS: lipopolysaccharides
MaR1: maresin 1
TRAP: tartrate resistant acid phosphatase
TV: total volume
VCAM: vascular cell adhesion molecule
VEGF: vascular endothelial growth factor
Veh: vehicle
VK: Von Kossa
