# Supplementary material for: Maresin 1 resolves aged-associated macrophage inflammation to improve bone regeneration
Source: FASEB J. Author manuscript; Available in PMC 2021 Oct 1. (PMC7719599; doi:10.1096/fj.202001145R)
Supplement: Supplementary Figures [file NIHMS1646586-supplement-Supplementary_Figures.pdf]

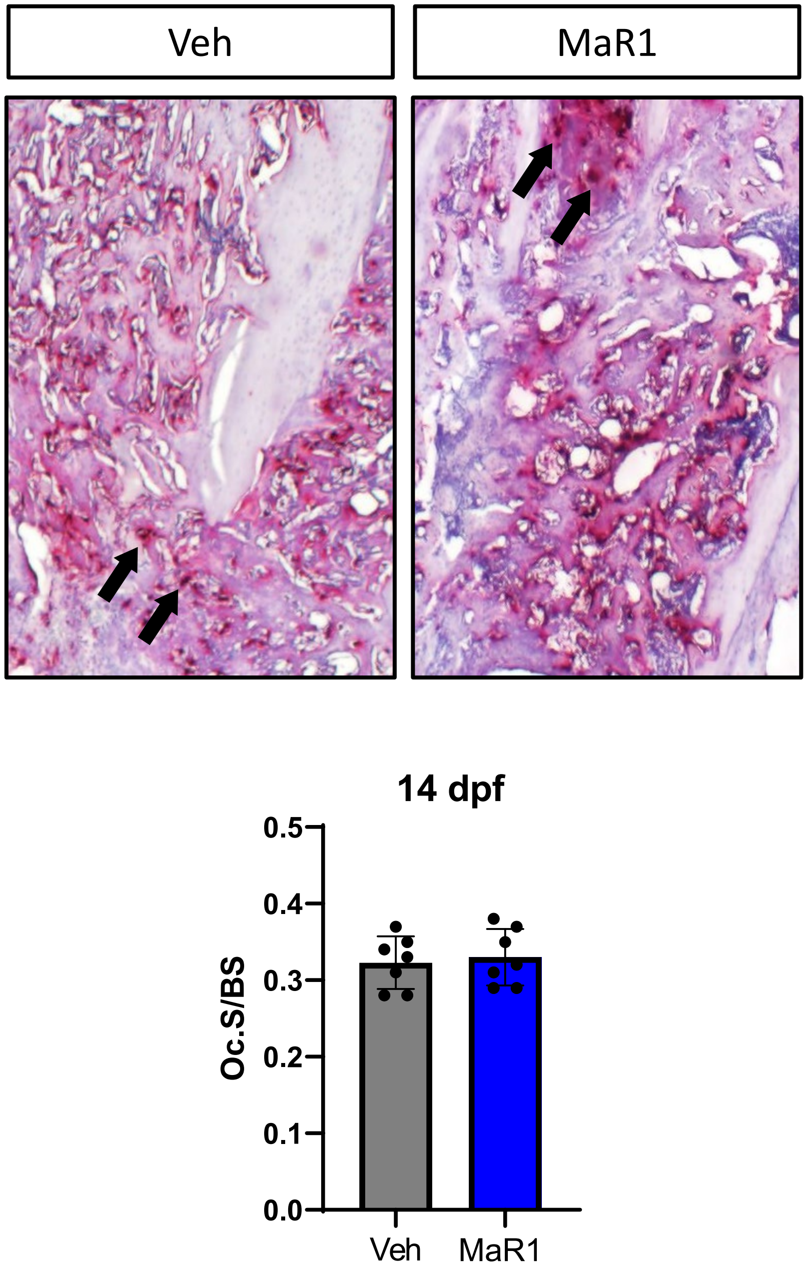

Supplemental Figure 1 – Mice were treated with either Veh or MaR1 3 days after fracture and calluses were stained for TRAP (top) and osteoclasts were quantified relative to bone surface (bottom).

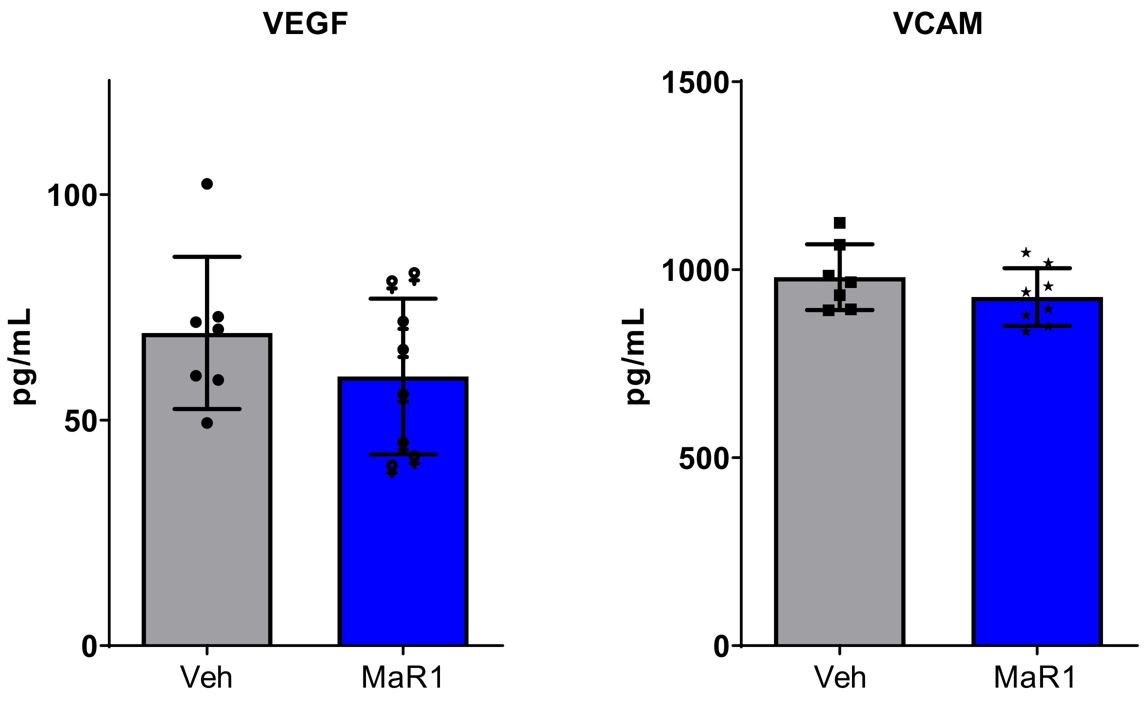

Supplemental Figure 2 – Mice were treated with either Veh or MaR1 3 days after fracture injury. After 7 days of healing, serum was collected and tested for levels of VEGF and VCAM by ELISA.

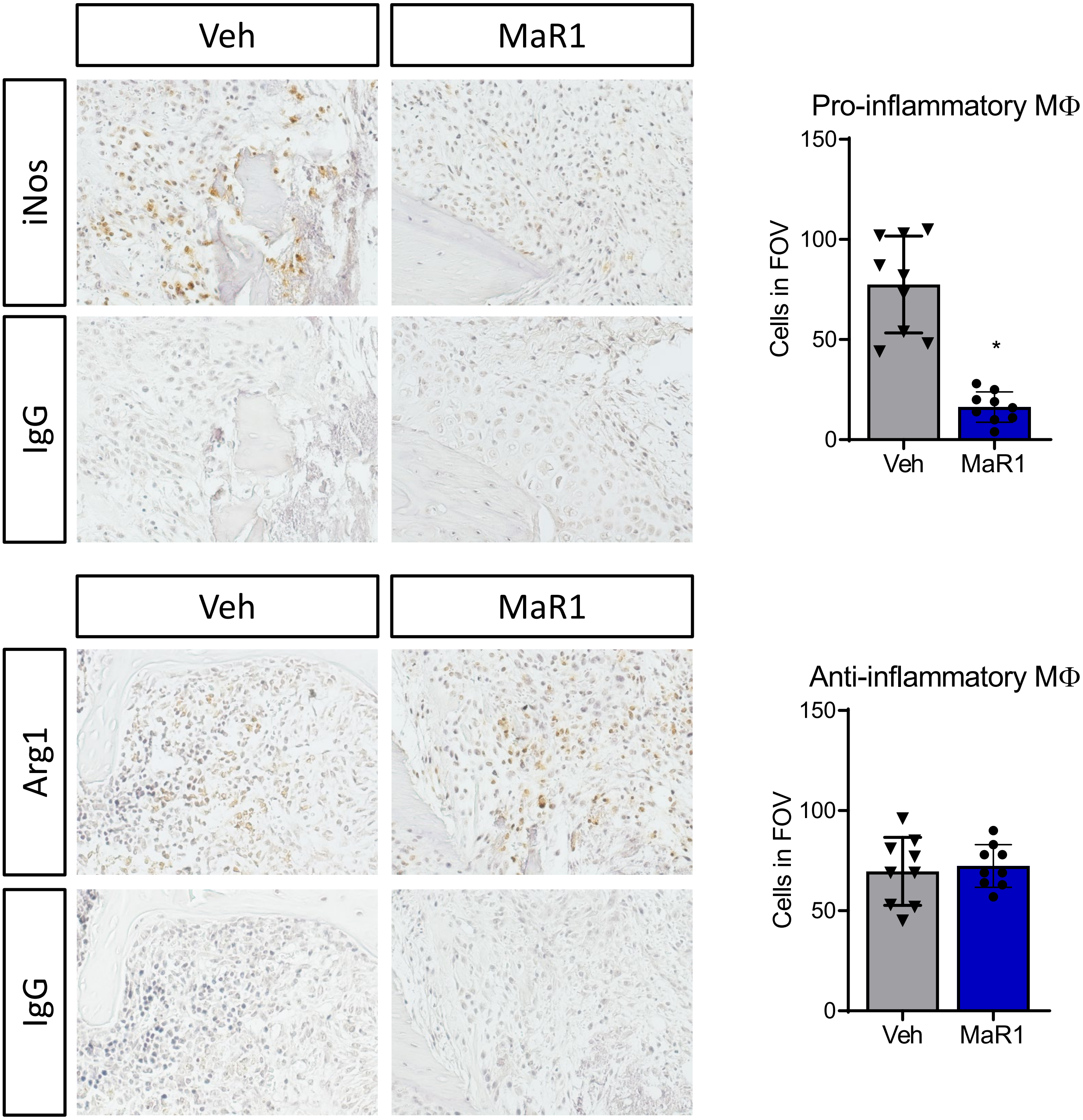

Supplemental Figure 3 – Mice were treated with either Veh or MaR1 3 days after fracture injury. 7-day fracture calluses were investigated using immunohistochemistry for iNos and Arg1. The number of positive cells per field of view were quantified.

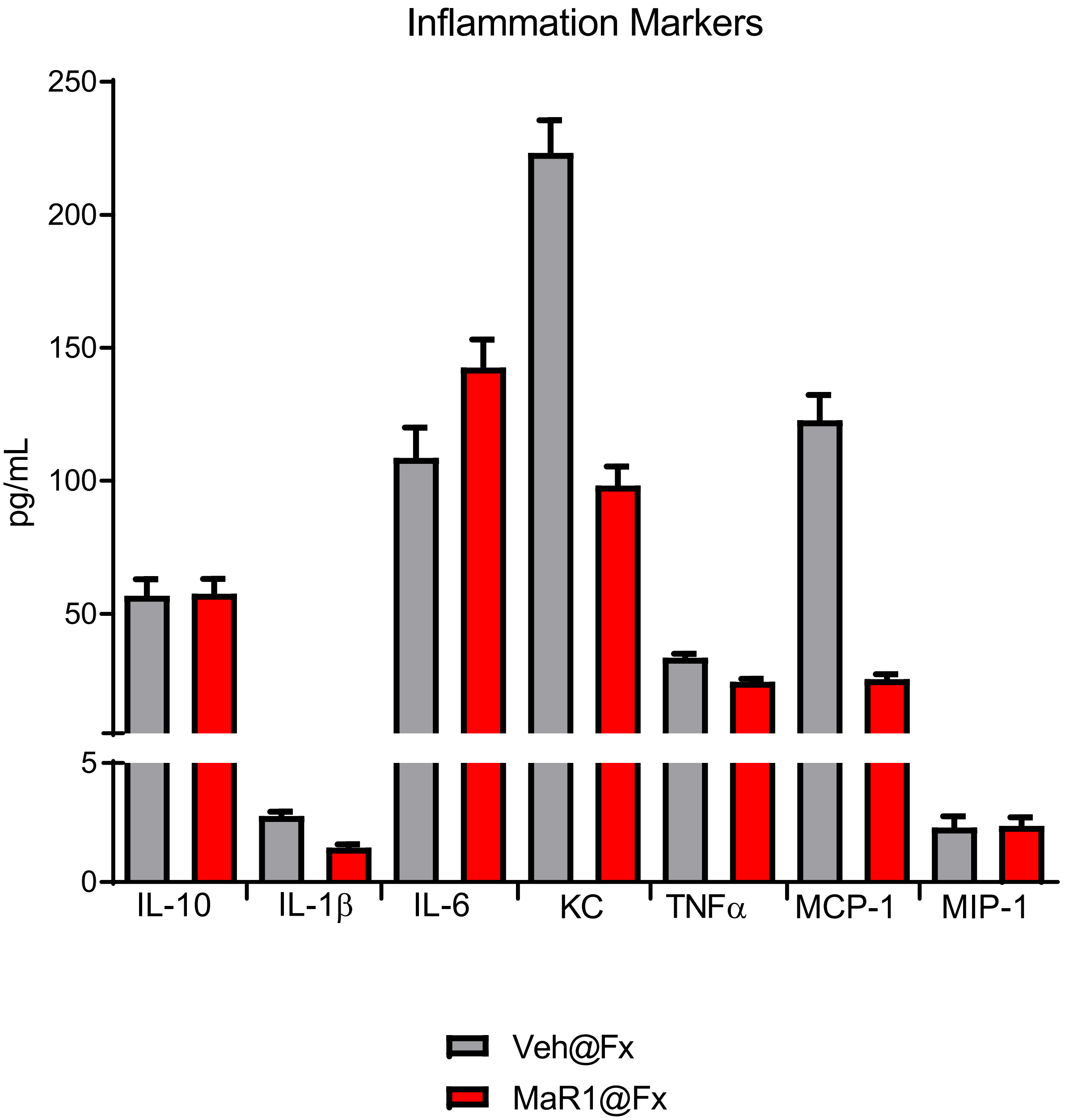

Supplemental Figure 4 – Mice were treated with either Veh or MaR1 at the time of fracture. After 7 days of healing, serum was collected and tested for levels of inflammatory biomarkers.

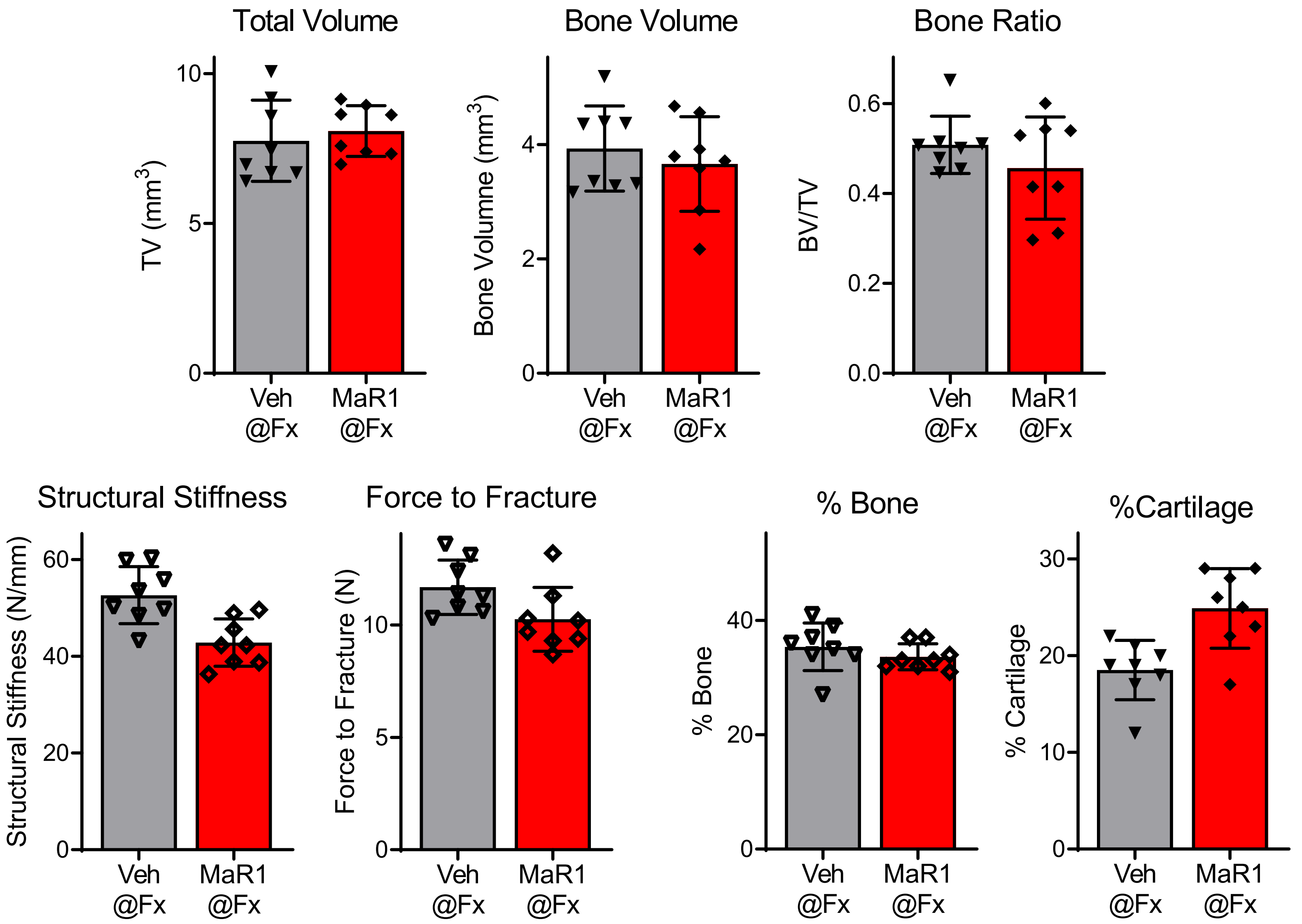

Supplemental Figure 5 – Mice were treated with either Veh or MaR1 at the time of fracture. (top) After 21days of healing, µCT analysis was used to determine Total Volume, Bone Volume, and Bone Ratio within the fracture callus. (bottom, left) After 28 days of healing, mechanical testing was used to determine the Structural Stiffness and the Force to Fracture of the healing tibia. (bottom, right) Histological analysis of 21-day fracture calluses was used to determine the % Bone and histological analysis of 14-day fracture calluses was used to determine the % Cartilage.

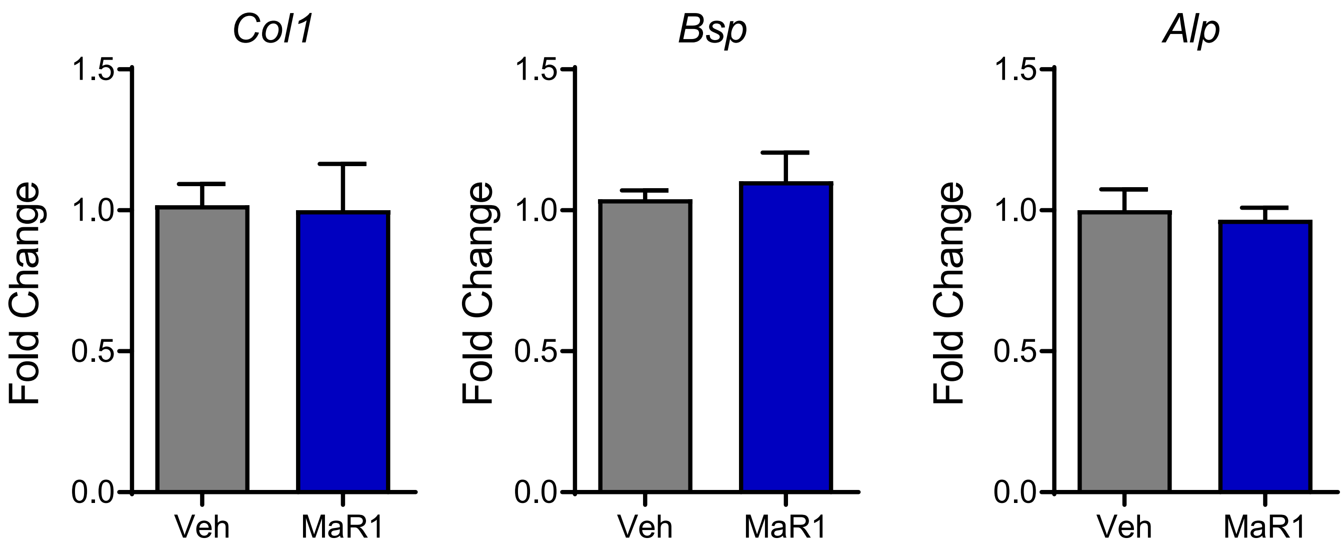

Supplemental Figure 6 – BMSC cultures were directly treated with Veh or MaR1 during osteoblastic differentiation. RNA was isolated 10 days after differentiation media was added and osteogenic transcripts were assessed using RT-PCR.
